# Arabinogalactan Proteins as Interactors along the Crosstalk between the Pollen Tube and the Female Tissues

**DOI:** 10.3389/fpls.2016.01895

**Published:** 2016-12-16

**Authors:** Ana M. Pereira, Ana L. Lopes, Sílvia Coimbra

**Affiliations:** ^1^Departamento de Biologia, Faculdade de Ciências da Universidade do PortoPorto, Portugal; ^2^Biosystems and Integrative Sciences InstitutePorto, Portugal

**Keywords:** plant reproduction, arabinogalactan proteins, pollen tube guidance, signaling, double fertilization

## Abstract

Arabinogalactan proteins (AGPs) have long been considered to be implicated in several steps of the reproductive process of flowering plants. Pollen tube growth along the pistil tissues requires a multiplicity of signaling pathways to be activated and turned off precisely, at crucial timepoints, to guarantee successful fertilization and seed production. In the recent years, an outstanding effort has been made by the plant reproduction scientific community in order to better understand this process. This resulted in the discovery of a fairly substantial number of new players essential for reproduction, as well as their modes of action and interactions. Besides all the indications of AGPs involvement in reproduction, there were no convincing evidences about it. Recently, several studies came out to prove what had long been suggested about this complex family of glycoproteins. AGPs consist of a large family of hydroxyproline-rich proteins, predicted to be anchored to the plasma membrane and extremely rich in sugars. These two last characteristics always made them perfect candidates to be involved in signaling mechanisms, in several plant developmental processes. New findings finally relate AGPs to concrete functions in plant reproduction. In this review, it is intended not only to describe how different molecules and signaling pathways are functioning to achieve fertilization, but also to integrate the recent discoveries about AGPs along this process.

## Arabinogalactan Proteins – The Forgotten Ones

The importance of the reproductive process for seed formation in flowering plants is, nowadays, unquestionable. This is a complex mechanism involving a series of signaling pathways with plenteous of well described players. The advances, made by live cell imaging and microscopy techniques allowed for a better understanding about how fertilization occurs. But classical reverse and forward genetics studies, microscopy ultrastructural analysis as well as the analyses of big data from microarrays or RNA-sequencing are still indispensable techniques for the discovery of new players and interactors with the already known molecules.

A big family of proteins that was always suggested to be involved in the reproductive process, since the formation of the gametophytes until fertilization is the Arabinogalactan protein (AGP) family. More recently, their functions in reproduction started to be unraveled, as well as their enigmatic mode of action. AGPs are a big family of hydroxyproline-rich glycoproteins, predicted to be tethered to the plasma membrane by a glycosylphosphatidylinositol (GPI) anchor (Schultz et al., 2002; Borner et al., 2003; Nguema-Ona et al., 2014). AGPs are composed primarily of carbohydrates surrounding a small proteinaceous core (Seifert and Roberts, 2007). These glycoproteins are suggested to play important functions in plants as signaling molecules given their particular characteristics: (1) GPI-anchor- which is a possible mode of release of these molecules to the extracellular medium where they may exert their functions, similar to what happens with the glycoprotein SKU5 (Sedbrook et al., 2002); (2) the high diversity of glycans decorating AGPs are also strong candidates to be released by proper enzymes and act as signaling molecules themselves. Sugar signaling in plants is only starting to be elucidated, much studies are required to fully understand how they act and are integrated in the several signal transduction pathways (Li and Sheen, 2016).

Recently Showalter et al. (2010) conducted a bioinformatic analysis identifying 85 AGPs in Arabidopsis, belonging to different sub-groups. According to their amino acidic composition AGPs can be classified as classical AGPs: Lysine-rich classical AGPs and arabinogalactan (AG) peptides; and chimeric AGPs: fasciclin-like AGPs (FLAs), plastocyanin AGPs, and other chimeric AGPs.

Several studies, in the most recent years, have contributed to unravel the mysterious mode of action of AGPs, supporting the hypothesis that AGPs carbohydrate components are AGPs’ main interacting and active constituents (Basu et al., 2013, 2015a,b; Ogawa-Ohnishi and Matsubayashi, 2015).

In these review we describe not only some of the already known molecules involved in plant reproduction, but we also integrate AGPs findings along the different phases of this process.

## Rising the Foundations of A Love Story

Double fertilization is a key process for successful development of a seed and the establishment of a new plant generation. It is a unique process, characteristic of Angiosperms, where two fertilizations occur simultaneously. This process was first described by S. Nawaschin in *Lilium martagon* and *Fritillaria tenella* in 1898. L. Guinard has confirmed the same phenomenon 1 year later, in 1899, independently, in *L. martagon* e *L. pyrenaicum* (Raghavan, 2003).

**FIGURE 1:**
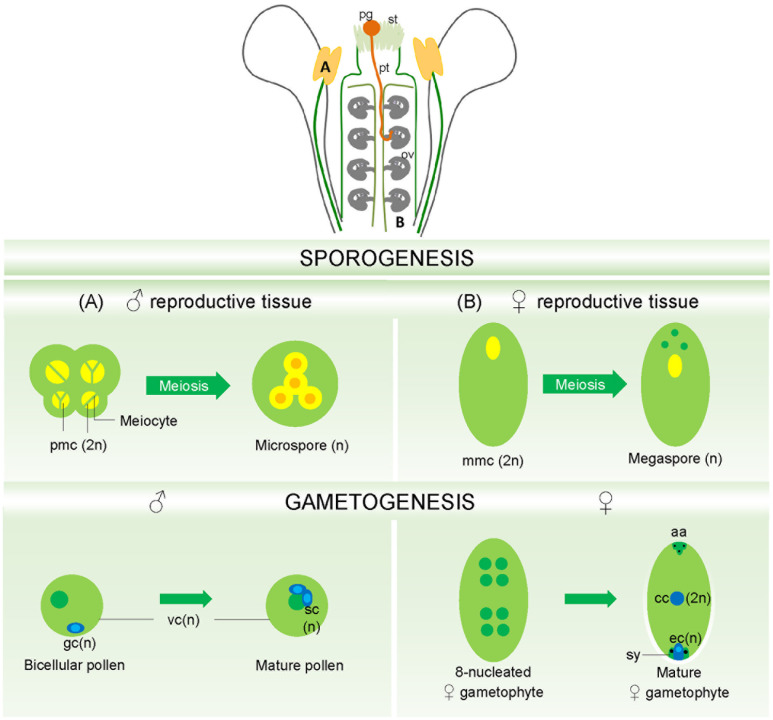
**Mature flower showing in detail male and female gametophyte’s formation.** **(A)** The pollen mother cells, located inside the anther locules, undergo meiosis generating the haploid microspores in a process called sporogenesis. Sporogenesis is followed by gametogenesis, where a mature pollen is formed, containing a vegetative cell engulfing two sperm cells, the male gametes. **(B)** The megaspore mother cells embedded in the ovular nucellar tissues also undergoes meiosis generating four haploid megaspores. From these, only one will survive and become the functional megaspore, which, after three rounds of mitosis and cellularization will give rise to the mature female gametophyte, the embryo sac. ov, ovule; pg, pollen grain; pt, pollen tube; st, stigma; pmc, pollen mother cell; mmc, megaspore mother cell; gc, generative cells; vc, vegetative cells; sc, sperm cells; cc, central cell; ec, egg cell; sy, synergids; aa, antipodals. (Mature flower reproduced with permission from Pereira et al., 2014).

In flowering plants, the male gametophyte develops inside the anthers locules (**Figure 1A**), part of the stamen, the male reproductive organ. It is released as a mature pollen grain containing a vegetative cell that will develop into a pollen tube and a generative cell that will generate by mitosis two sperm cells: the male gametes (McCormick, 2004; Borg and Twell, 2010).

The female gametophyte develops inside the ovule, deeply embedded in the sporophytic tissues of the pistil (**Figure 1B**). During megasporogenesis a cell from the nucellus, the archesporial cell, differentiates into the megaspore mother cell, which undergoes meiosis giving rise to four haploid megaspores. One of these megaspores develops into the functional megaspore and the remaining three megaspores will undergo programmed cell death (PCD). In the *Polygonum*-type of female gametophyte (Yadegari and Drews, 2004), the functional megaspore undertakes three rounds of nuclear divisions (mitosis without cytokinesis) resulting in a coenocyte with eight nuclei at distinct positions of the female gametophyte. After migration of the nuclei and cellularization, the mature embryo sac or female gametophyte is formed, a seven celled, eight-nucleated structure that comprises: three antipodal cells positioned in the chalazal pole of the ovule, one diploid central cell and two synergid cells surrounding the egg cell, at the micropylar pole of the ovule. Here are kept the two female gametes: the central cell and the egg cell (Huang and Russell, 1992; Drews et al., 1998; Yadegari and Drews, 2004).

Pennell and Roberts (1990) had foreseen that in *Pisum sativum*, the switch from a vegetative to a reproductive stage of development was related with the presence or absence of arabinose-containing epitopes identified by the monoclonal antibody MAC207 (Pennell et al., 1989), describing these AGPs epitopes only in the sporophytic tissues. Later, a new monoclonal antibody characterized by Pennell et al. (1991), JIM8, recognized AGP sugar epitopes also during the gametophytic phase of development: in sperm cells, egg cells and in the synergids filiform apparatus in *Brassica napus* flowers. Coimbra et al. (2007) using several anti-AGP antibodies, identified AGP-specific sugar epitopes in the functional megaspore and in the female gametophyte: synergid cells and in the synergid filiform apparatus of Arabidopsis, confirming the previous immunolocalization studies and the concept of differential expression of AGPs in plant reproductive tissues.

Later, AGP18 was identified as an AGP essential for female gametophyte formation (Acosta-García and Vielle-Calzada, 2004; Demesa-Arévalo and Vielle-Calzada, 2013). AGP18 was shown to be transcribed specially in the megaspore mother cell, but its glycosylation pattern was only defined in the functional megaspore, confirming the importance of AGPs in cell fate determination. This also revealed that the full activation of AGPs function in diferent tissues is dependent on the presence of specific glycosylation enzymes at particular developmental timings, and pinpoint their importance as potential cell fate determination factors as suggested by Pennell et al. (1991). Tucker et al. (2012) and Tucker and Koltunow (2014) also focused on the importance of other AGPs such as AGP22 and AGP24, in the megaspore mother cell and in the functional megaspore formation and development. Yellow fluorescent protein expression driven by AGP22 and AGP24 promoters targeted the nucellar epidermal cells and the functional megaspore, respectively, supporting the immunolocalization studies made with JIM13 by Coimbra et al. (2007), and their possible roles as cell fate determination factors.

Immunolocation has been widely used as the main tool to study AGPs localization (Pereira et al., 2015). Even if the antibodies are directed to the glycosidic moiety and do not recognize a specific AGP, they are still valuable tools to dissect functions, as demonstrated by the studies described above. These have shown the importance of AGPs in the differentiation of the functional megaspore, which gives rise to the female gametophyte. AGPs localization studies using classical molecular tools are still poorly explored due to the complexity of these molecules. The putative presence of a GPI anchor and the high amount of carbohydrate chains makes it difficult to obtain a stable construct expressing a reporter gene. This may lead to conformational changes that will affect the glycoprotein activity. So far, most of the AGPs localization studies with reporter genes such as the green fluorescent protein or the β-glucuronidase, have been made using only the respective AGP promoter region, allowing us to access only its localization in diferent tissues and cell types, but not their intracellular location (Pereira et al., 2014).

## Double Fertilization – Twosomes, A Love Story

When a mature pollen grain finally reaches the stigmatic cells and is recognized as compatible pollen, it adheres to the stigmatic cell and hydration begins, leading to the formation of a protrusion. This protrusion will become the pollen tube that is responsible for carrying the two sperm cells into the embryo sac. The fast and tightly controlled growth of the pollen tube tip is ensured by a series of communication and consequent signaling cascades, which result from several interactions between the pollen grain and the pollen tube with the female sporophytic and gametophytic tissues. Given this, the pollen tube tip grows along the extracellular matrix of the stylar and transmitting tract tissues until it perceives signals that will make it turn abruptly in the direction of an ovule. At this moment the pollen tube quickly turns into the placenta tissues, growing along the funiculus until it reaches the embryo sac entrance: the micropyle. Once in the micropylar region the pollen tube enters the embryo sac through the filiform apparatus of one of the two synergids, ceasing its growth, rupturing and releasing its two sperm cells. The invaded synergid dies after pollen tube entrance, and the persisting synergid will undergo synergid-endosperm fusion after successful fertilization of both female gametes. The sperm cells migrate to the egg and central cell, and fuse with them, giving rise, respectively, to the embryo and the nourishing tissue, the endosperm, finally accomplishing double fertilization (reviewed in Palanivelu and Tsukamoto, 2012; Beale and Johnson, 2013; Dresselhaus and Franklin-Tong, 2013).

Recently, the knowledge developed on peptide signaling became crucial to understand the molecular mechanisms along the pollen tube journey and during double fertilization. Secreted peptides of various cysteine-rich peptides (CRPs) subclasses were identified as players in pollen grain recognition at the stigma, in pollen tube growth support and guidance, in attracting the tube to enter the embryo sac and in gamete interaction mediation (Qu et al., 2015).

Large gene families of peptides are involved in double fertilization and most members show similar expression patterns with redundant function, as shown in studies of EC1s (Sprunck et al., 2012), LUREs (Takeuchi and Higashiyama, 2012), and ES1–4 (Amien et al., 2010; Woriedh et al., 2015). CRP subfamilies like LTPs (lipid-transfer proteins) and RALFs (RAPID ALKALINIZATION FACTOR) are strongly expressed along the pollen tube pathway, and some functions are starting to be revealed. As indicated by Bircheneder and Dresselhaus (2016), RALF peptides secreted from the pollen tube may act as pollen-derived interaction partners of synergid-localized FER (FERONIA) to induce male-female communication events. Like this, other intensive communication takes place during the pollen tube journey within the maternal tissues of the stigma, style, transmitting tract and ovule. These processes have been recently fairly reviewed (Palanivelu and Tsukamoto, 2012; Dresselhaus and Franklin-Tong, 2013; Bleckmann et al., 2014; Higashiyama and Takeuchi, 2015; Qu et al., 2015; Dresselhaus et al., 2016) although not enough attention was given to AGPs as basal component interactors.

The following sections highlight several communication steps between the male gametophyte and the female reproductive tissues: (i) interaction events during the growth of the pollen tube throughout the female flower tissues, (ii) molecular mechanisms during pollen tube reception, and (iii) double fertilization and multiple pollen tube blockage.

## Pollen Tube Germination and Growth – The Love Story Begins

Pollen tube growth from the stigma until the embryo sac may be divided in two different phases:

- A sporophytic phase, that comprises pollen tube growth from the stigma, through the style and the transmitting tract, and independent from molecular cues from the FG;
- gametophytic phase, which depends also on molecular cues provided by the FG, and refers to pollen tube growth from the moment it makes a quick turn into the septum surface and enters the funiculus targeting the micropyle of the embryo sac.

The sprorophytic phase starts with pollen grain in the stigma, the initial adhesion of the pollen grain to the stigma surface is largely dependent on the outer wall of the pollen grain, the exine (Zinkl et al., 1999). Immediately after contact with stigmatic cells, the pollen coat is extruded of the exine wall against the stigma surface, forming a ‘foot’ that strongly sticks to the stigma (Chapman and Goring, 2010). Hydration of the pollen grain follows this step, depending on the pollen coat lipids to control the movement of water from the stigma (Wolters-Arts et al., 1998; Mayfield and Preuss, 2000; Mayfield et al., 2001).

Though the pollen grain has great responsibility in initiating compatible pollen grain germination in the stigma (reviewed in Doucet et al., 2016), its female partner is also essential for this successful interaction. Exo70A1, an exocyst complex subunit present in *Arabidopsis thaliana* stigmas is needed for acceptance of compatible pollen grain. Exo70A1 is proposed to act in the polarized secretion of stigmatic cells to deliver vesicles containing aquaporins for increased water permeability, allowing pollen hydration, as well as cell wall–modifying enzymes, to allow pollen tube penetration through the stigma (Samuel et al., 2009). Through the study of mutants with reduced levels of phosphatidylinositol-4-phosphate (PI4P) in the stigma, PI4P was also shown to be essential for this initial step of pollen–pistil interaction. These mutants revealed slower rates of pollen grain hydration and showed maternal fertility defects due to a higher level of failed pollinations (Chapman and Goring, 2011). This study suggested that phosphoinositides, which are important lipids involved in polarized secretion in general, have a specific role in pollen hydration. Although in animals and yeast, the Exo70A1 is proposed to bind PIP4, 5P_2_ (He et al., 2007; Liu et al., 2007) defining the location of the exocyst assembly, this study didn’t show the same mode of action in plants.

Pollen tube-female tissues interactions entail intercellular communication between different cell types, requiring the compartmentalization and degradation of signaling molecules and, many times, the continuation or starting of the signaling pathway after internalization, as well as the recycling of cell wall molecules, via the endocytic pathway. An *A. thaliana* Vacuolar Protein Sorting 41 (AtVPS41) was very recently identified as being a new factor controlling pollen tube-stigma interaction (Hao et al., 2016). *Atvps41* pollen tubes fail to penetrate the female transmitting tract due to an impairment in the endocytic pathway.

Arabinogalactan proteins are present in the stigma cells and in the growing pollen tubes, thus suggesting possible roles for them in pollen–stigma cells interactions, and acquisition of pollen grain competence to initiate pollen tube growth. AGP6 and AGP11, pollen specific AGPs, were shown to be involved in the pollen tube endosome machinery (Costa et al., 2013). Yeast-2-Hybrid experiments made with these two AGPs showed their physiological interaction with several members of the endosomal system. As suggested in the above studies, the pollen tube interactions with the female tissues requires constant integration of signaling molecules from the extracellular matrix, degradation and/or recycling of these molecules, and in the case of AGPs, their internalization and secretion through multivesicular bodies (MVBs) (Costa et al., 2013). This is coherent with Pereira et al. (2006) results, where the site of pollen tube emergence was labeled with MAC207 by immunolocalization, an antibody that recognizes AGPs carbohydrate epitopes. This antibody labeled the growing pollen tube after germination. When using a different antibody, LM2, which recognizes other AGPs specific carbohydrates, the labeling was different, restricted to a “collar-like” shape in the emerging pollen tube. Dardelle et al. (2010) confirmed these results, showing that LM2 also has a stronger labeling at the pollent tube tip, where a highly dynamic intracellular trafficking occurs targeting the transport of vesicles. Clearly, polar pollen tube growth involves the integration of several signal transduction pathways through the membrane and the cell wall, where AGPs appear to be involved, greatly controlled by the regulation of vesicle trafficking.

Ca^2+^ is an important signaling molecule during pollen tube growth along the pistil tissues. Monitoring of Ca^2+^ dynamics during pollination both in pollen grain and stigmatic cells revealed some interesting results (Iwano et al., 2004). The cytosolic [Ca^2+^] increased at different time points: in pollen grain, after hydration until germination, and in the stigmatic cells after pollen grain adhesion and hydration, after pollen tube emergence from the pollen grain and after the pollen tube penetrated the stigmatic cell wall. This shows that Ca^2+^ acts most probably as an intracellular signaling factor during pollen grain and stigmatic cells interaction, for pollen tube formation and initial polarized tip growth. Curiously, recent findings about the enigmatic mode of action of AGPs, suggested they might act as Ca^2+^ capacitors (Lamport and Várnai, 2012; Lamport et al., 2014). If so, AGPs may be important molecules in this first steps of interaction, being responsible for the release of periplasmic Ca^2+^ to be integrated in these cytosolic Ca^2+^ oscilations.

By using immunolocalization studies Losada and Herrero (2012) discovered that the developmental pattern of AGP expression in stigmatic tissues of the apple flower were correlated with the stigma receptivity stages, supporting the above hypothesis of AGPs involvement in this important step of plant reproduction. Later, the same authors (Losada and Herrero, 2014) also showed that, opposed to what happens in Arabidopsis, in *Mallus domestica* AGPs are detected in the stigma but not in the transmitting tract, and this relates to the fact that pollen tubes grow slower in the stigmatic tissues and faster in the transmitting tract tissues.

Finally, after initiating the growth through the stigmatic cell walls, the pollen tube will start a long journey that will lead it into the embryo sac.

## Pollen Tube Growth on the Superhighway – Courtship

Once germinated and the pollen tube formed, a new phase in the pollen tube–pistil interaction story starts. In Arabidopsis pollen tubes penetrate stigmatic cells and grow directly through the cell wall and into the cytosol, forcing their way through the basal wall region and into the transmitting tract tissue (Hiscock and Allen, 2008). The pollen tube has an intercellular growth through the stylar cells until reaching the embryo sac (Hulskamp et al., 1995; Johnson and Preuss, 2002; Lord and Russell, 2002).

In the initial sporophytic phase of attraction, the pollen tube grows through the transmitting tract, a specialized tissue extremely rich in glycoproteins, polysaccharides, and glycolipids, which are thought to facilitate and nourish the pollen tube development (Faure et al., 2002; Crawford and Yanofsky, 2008).

In *Nicotiana tabacum* AGPs called TTS (Transmitting Tissue-Specific) are abundant in the extracellular matrix of the transmitting tract, being related to pollen tube guidance into the ovules. These glycoproteins stimulate pollen tube growth *in vitro*, are able to attract pollen tubes in *semi-in vivo* assays, and are essential for optimal pollen tube growth *in vivo*. The action of pollen tube hydrolases on the carbohydrate component of these glycoproteins conduces to the establishement of an increasing gradient from the top to the bottom of Nicotiana styles, which is suggested to have a chemotropic effect on growing pollen tubes. TTS are proposed to function as a nutrient source and act as adhesion molecules for pollen tube growth (Cheung et al., 1995; Wu et al., 1995, 2001). Arabidopsis *ntt* (*no transmitting tract*) mutants, with abnormal transmitting tract development, show severe defects in pollen tube growth, which is slower, leading to a reduced fertilization rate (Crawford et al., 2007). *NTT* encodes a C2H2/C2HC zinc finger transcription factor involved in the control of extracellular matrix production, being essential for PCD in transmitting tract tissues after pollination, essential for pollen tube growth (Crawford et al., 2007). Besides, the *ntt* mutants presents a reduced staining for acidic polysaccharides, which Crawford et al. (2007) speculated to be related to a reduction in AGPs content, since these are acidic glycoproteins and a main component of the transmitting tract. In fact, specific AGPs such as AGP1, AGP4 (JAGGER), and AGP19, were shown to be abundantly expressed in this tissue (Yang et al., 2007; Pereira et al., 2014, 2016b). JAGGER was identified as an AGP highly expressed in the transmitting tract tissues (Pereira et al., 2016b) although its mutant analysis did not reveal any phenotype related to this expression pattern. It will be of most interest to check whether transmitting tract AGPs expression is somehow under the control of the NTT transcription factor, and other specific transmitting tract transcription factors, such as the HECATE1, HECATE2, and HECATE3 (Gremski et al., 2007).

**FIGURE 2:**
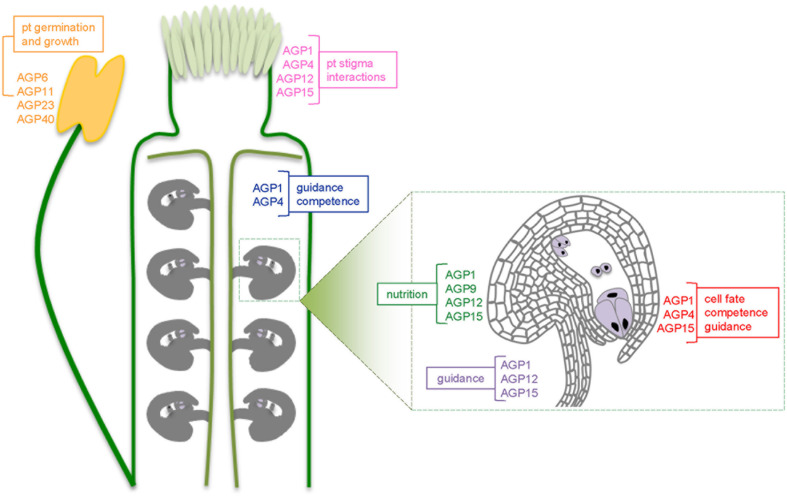
**Schematic representation of an Arabidopis pistil and, in more detail, one ovule, showing the different AGPs present along the pollen tube growth pathway.** In pink are represented the AGPs from the stigma and the pistil: AGP1, AGP4, AGP12, and AGP15; in blue the ones known to be expressed in the transmitting tract: AGP1 and AGP4. The ovule scheme shows in green the AGPs in the chalaza: AGP1, AGP9, AGP12, and AGP15, in red AGPs present along the integuments near the micropyle: AGP1, AGP4, and AGP15 and in violet in the funiculus: AGP1, AGP12, and AGP15. For each sub-group of AGPs it’s highlighted its possible functions.

Several AGPs are already identified along the whole pollen tube growth pathway (**Figure 2**). AGP1, 4, 12, and 15 were identified in papilla cells of the stigma and in the style, and may be responsible for the functions proposed for AGPs in the above section. AGP1 and AGP4 in transmitting tract cells of the style and in the ovary, while AGP1, AGP4, AGP9, AGP12, and AGP15 are expressed in ovules, which develop into seeds after fertilization (Pereira et al., 2014). The genetic expression and function of another AGP, AGP19, was characterized by Yang et al. (2007) and the GUS promoter analysis showed its expression in the style, ovary walls, transmitting tract and siliques, with no expression in anthers, indicating its specificity to the female tissues. Its knockout plants revealed reduced flower production and fewer and shorter siliques than wild-type plants, revealing the importance of this specific AGP in reproduction. All these studies point out the importance of AGPs along the reproductive process.

Other important players have been shown to be involved in the molecular interactions between the pollen tube and the transmitting tract tissues. *POLLEN ON PISTIL 2* (POP2) encoding a γ-aminobutyric acid (GABA) transaminase, is involved in degrading GABA, establishing an increasing [GABA] gradient from the stigma until the ovule integuments, similar to what happens with the TTS glycoproteins (Palanivelu et al., 2003). This gradient is also believed to sustain pollen tube guidance though the transmitting tract. A plant lipid transfer protein, SCA (stylar cysteine-rich adhesion), was shown to be secreted from the transmitting tract epidermis and involved in adhesion-mediated pollen tube guidance by the formation of an adhesive pectin matrix that guides the pollen tubes toward the ovules (Mollet et al., 2000; Park et al., 2000). Interestingly, one recently identified AGP, AGP57C (Showalter et al., 2010) was shown to be the core of a proteoglycan with covalently attached arabinogalactan, arabinoxylan, and pectin domains. This highly glycosylated proteoglycan was named ARABINOXYLAN PECTIN ARABINOGALACTAN PROTEIN1 (APAP1) by Tan et al. (2013). This AGP was shown to cross-link in cell walls with pectin and hemicellulose polysaccharides, allowing the formation of a continuous network between polysaccharides and wall structural proteins. This APAP1 may be involved in the formation of the adhesive pectin matrix proposed earlier for pollen tube guidance mediation (Mollet et al., 2000; Park et al., 2000).

In *L. longiflorum* a chemotropic compound from the stigma was identified as a chemocyanin. This small plantacyanin cell wall protein, appears to regulate lily pollen tube directional growth (Kim et al., 2003). In *A. thaliana*, a unique plantacyanin gene was found with 51.9% identity and 86.8% similarity to lily chemocyanin at the amino acid level. Dong et al. (2005) hypothesized that this plantacyanin may act in the reproductive process, since it is part of the extracellular matrix of the transmitting tract and its overexpression in the pistil led to the disruption of pollen tube growth from the stigma to the style. Later, Mashiguchi et al. (2009) identified a family of early nodulin-like proteins (ENODLs) as chimeric AGPs, related to the phytocyanin family. In this study, the characterization of single ENODLs mutants did not reveal any novel biological functions for them, despite their high expression in floral organs. This ENODLs share AGPs characteristics, binding to the β-Yariv reagent, a synthetic chemical that specifically bind to AGPs. But also share a structure similarity with phytocyanins, although they lack the specific sub-unit that binds copper, like most phytocyanins. Showalter et al. (2010) have also identified this ENODLs as chimeric AGPs. But only now, their functions started to be unraveled by Hou et al. (2016), as it will be described in the next section.

More recently, the amino acid *D*-serine was shown to play a role in pollen tube guidance through the transmitting tract by mediating Ca^2+^-influx into the pollen tube cytoplasm involving glutamate receptor–like channels (Michard et al., 2011). Once again, such as in stigma-pollen grain interactions, AGPs may serve as Ca^2+^ capacitors (Lamport and Várnai, 2012; Lamport et al., 2014), making Ca^2+^ available for its influx mediated by *D*-serine.

Still, it is not clear how all these transmitting tract constituents interact with the growing pollen tube promoting or/and supporting its growth. It is clear that there must be a tightly controlled activation and/or inactivation of specific receptors in pollen tubes and transmitting tract cells as well as the production of ligands by the transmitting tract to coordinate the pollen tube growth toward the embryo sac. Several microarray-based approaches have been used to identify novel intervenients in pollen tube-sporophyte interaction mechanisms (Tung et al., 2005; Qin et al., 2009; Boavida et al., 2011). Interestingly, all these studies show enrichment in secreted proteins and cell-wall-related proteins potentially involved in extracellular signaling and extracellular matrix modifications. Besides the importance of these interactions for the pollen tube growth along the pistil tissues, the different components present in these tissues point out for a contact-mediated competence that must be conferred by the stigma and style to the pollen tube in order for it to become receptive to ovule signals (Palanivelu and Preuss, 2006).

The presence of AGPs along this pollen tube pathway into the ovule embryo sac has been documented in numerous and phylogenetically diverse plant species [Gleeson and Clarke, 1980; Hoggart and Clarke, 1984; Sedgley et al., 1985; Webb and Williams, 1988; Jauh and Lord, 1996; Coimbra and Salema, 1997; Lennon et al., 1998; Cheung et al., 2000; Wu et al., 2000; Coimbra and Duarte, 2003; Peng et al., 2005; Coimbra et al., 2007; Losada and Herrero, 2012, 2014; Costa et al., 2013; Suárez et al., 2013 (for a more detailed description see Pereira et al., 2015)].

A new study on pollen tube guidance discovered AMOR, a sporophytic ovular factor that induces pollen tube competency in Torenia to respond to female signals (Mizukami et al., 2016). Mizukami et al. (2016) discovered a non-proteinaceous competence factor, acting in a non-species-preferential manner. AMOR activity was identified as a methyl-glucuronosyl arabinogalactan (AG) polysaccharide. A chemically synthesized disaccharide, the β-epimer of methyl-glucuronosyl galactose (4-Me-GlcA-β-(1/6)-Gal or 4Me-GlcA-Gal), showed full AMOR activity, while the disaccharide lacking a methyl group was completely inactive. The disaccharide 4Me-GlcA-Gal occurs exposed on AGPs side chains (Pereira et al., 2015). Arabinogalactan polysaccharides are added to proteins using *O*-glycosylation onto hydroxyproline residues in the AGP backbone – the addition of this polysaccharides results in a glycoprotein with a mainly carbohydrate interactive molecular surface. In Torenia, the arabinogalactan AMOR enables pollen tubes to respond to female gametophyte-derived attraction signals. Notably, AGPs are very abundant along the pollen tube pathway and occur in the transmitting tract, placenta, funiculus and the micropylar opening of the ovule in Arabidopsis and many other species as mentioned above (Cheung et al., 1995; Coimbra et al., 2007; Pereira et al., 2014). These AGPs are most probably the source of this AMOR glycan. What remains to be determined is which specific AGP is responsible for their production? How are they “cleaved” out of the AGP and released into the extracellular medium? Which enzymes act in this possible step? Besides all these unanswered questions, the most important now is to know what happens in the model species *A. thaliana*, because, as Dresselhaus and Coimbra (2016) reminded us “pollen tubes, for example, appear almost competent without ovules and thus may not need priming by AMOR.” However, in Arabidopsis it is known that pollen tubes grown in semi-*in vivo* assays, as long as they pass through a cut style, they become competent enough to reach the ovules and enter it (Palanivelu and Preuss, 2006). And here, another question arises: since these tissues (stigma, and style transmitting tract) are extremely rich in AGPs, can it be that they have already become competent because they were in contact with enough AMOR glycans? Or this step does not involve AGPs, but other molecules? This implies to better understand AGPs mode of action, which components of this complex hydroxyproline-rich proteins are essential for the pollen tubes to acquire competence, or even to act in other functions, such as nutrition or guidance.

## Funicular and Micropylar Growth – Sex Laws

Contrarily to the pollen tube guidance phase described above, completely dependent on signals from the sporophyte, the funicular and micropylar growth of the pollen tube is dependent on cues delivered, not only by the sporophytic tissues, but also by the female gametophyte. The Arabidopsis mutants *magatama* (*maa1* and *maa3*, the latter encoding a helicase) show an abnormal female gametophyte development with pollen tubes being targeted to the micropylar region, but not entering the synergids (Shimizu and Okada, 2000; Shimizu et al., 2008). Also in Arabidopsis, two predicted K^+^ transporters: CHX21 and CHX23 present in pollen tubes, are essential for pollen tube guidance at this stage. *chx21* and *chx23* pollen tubes grow normally through the transmitting tract but fail to turn in the direction of the ovule and do not grow along the funiculus. The perception of some ovule signals that are critical to shifting the axis of pollen tube polarity and directing its growth toward the ovule most probably fail in these mutants (Lu et al., 2011). Mitogen-activated protein kinases MPK3 and MPK4 from pollen were additionally shown to be essential for pollen tube guidance during the funicular guidance phase, although the micropylar guidance was not affected (Guan et al., 2014). A most recent study revealed that phytosulfokine (PSK) is essential to guide the pollen tube from the transmitting tract to the embryo sac. PSK is perceived by receptor kinases and requires sulfation by a tyrosylprotein sulfotransferase (TPST) to be active. *pskr1-3 pskr2-1* and *tpst* siliques present a high level of unfertilized ovules and loss of funicular guidance, suggesting the importance of PSK for funicular guidance (Stührwohldt et al., 2015). Curiously, PSKR1 was recently shown to be involved in plant defense to pathogens as well as PSK (Mosher and Kemmerling, 2013). The MPK3/MPK6 signaling pathway and the PSK signaling may link common signaling networks in plant stress response and pollen–pistil interactions. AGPs have been identified in the funiculus of Arabidopsis plants, such as AGP1, AGP12, and AGP15 (Pereira et al., 2014). These glycoproteins may play important signaling roles along this step, where pollen tubes turn from the transmitting tract to grow along the funiculus. Clearly, pollen tubes require a multiplicity of signaling pathways in order to perceive and respond to ovule signaling and determine its growth direction.

Considerable progress has been made in the last years in order to better understand what controls the last phase of pollen tube guidance, the micropylar guidance phase. When pollen tubes arrive at the micropylar region they must grow through this opening between the integuments of the ovules to reach the filiform apparatus, which are invaginations from the synergids’ cell wall that greatly increase their extracellular contact area, as typical for active secreting cells (Huang and Russell, 1992). In *Torenia fournieri*, a species with a naked embryo sac, it was shown that the synergids are the female gametophyte cells responsible for the gametophytic guidance phase. Laser ablation studies revealed that both synergids are essential for pollen tube guidance, but a single synergid is also capable of attracting pollen tubes into the embryo sac (Higashiyama et al., 1998, 2001). Moreover, in Arabidopsis, the importance of the synergids as the source of guiding molecules in this phase was shown, by studying a transcription factor mutant, *myb98*. In this mutant the ovule develops normally, excepot for the synergids filiform apparatus, which does not develop normally, impairing pollen tube guidance at this step (Kasahara et al., 2005; Punwani and Drews, 2008). So, the synergid cells are the main source for chemo-attractants necessary for micropylar pollen tube guidance.

So far, molecular attractants produced by the synergid cells were identified in three different species. In *T. fournieri* a group of defensine like polypeptides (DEFL) was identified as pollen tube attractants produced by the synergids and secreted to the filiform apparatus’ surface, the LUREs (Okuda et al., 2009). A DEFL subgroup CRP810/*At*LURE1, was also identified in Arabidopsis, revealing to be essential for micropylar pollen tube guidance (Takeuchi and Higashiyama, 2012). In *Zea mays*, the EGG APPARATUS1 (*Zm*EA1), a small hydrophobic precursor protein of 94 aminoacids, is present not only in the synergids, but also in the egg cell, in the filiform apparatus and in the micropylar nucellus, and it is required for micropylar pollen tube guidance (Márton et al., 2005, 2012). The synergids are not the only cells regulating pollen tube guidance in *Arabidopsis*, *CENTRAL CELL GUIDANCE* (*CCG*) is expressed in the nucleus of the central cell and might function as a transcriptional regulator mediating pollen tube guidance, since pollen tubes fail to target the micropyle of mutant ovules (Chen et al., 2007). Furthermore, the egg cell expressed GEX3 protein was shown to play a role in ovular guidance of the pollen tube (Alandete-Saez et al., 2008).

On the male side two receptor-like kinases (RLKs) were shown to impair micropylar pollen tube guidance, LOST IN POLLEN TUBE GUIDANCE1 (LIP1) and 2 (LIP2). They are both localized in the pollen tube membrane and are involved in the AtLURE1-dependent guidance mechanism (Liu et al., 2013) although more evidences are necessary to prove that LIP1 and LIP2 interact with *At*LUREs. Recently, a receptor that recognizes AtLURE1 was identified in Arabidopsis, a heteromer MDIS1-MIK, composed by two pairs of closely related RLKs: Male Discoverer 1 and 2 (MDIS1 and MDIS2) and MDIS1-interacting RLK1 and 2 (MIK1 and MIK2) (Wang et al., 2016). All these molecules may act together as essential components of the receptor complex of pollen tube guidance, although the way in which this happens remains to be uncovered. Simultaneously, Takeuchi and Higashiyama (2016) discovered another set of pollen-specific RLK, PRKs, responsible for the sensing of AtLURE1 by the pollen tube. At the end, we have so many receptors, and only one attractant molecule in Arabidopsis? According to Higashiyama and Hamamura (2008) it is still unclear if there is only one attractant molecule produced by the synergids to guide the pollen tubes into the embryo sac or if several molecules are working redundantly or even together to control such an important step in plant reproduction. It is more likely that several molecules involved in an intricate signaling pathway are acting in this micropylar guidance phase, since, until today, there is no known mutant where a total blockage of pollen tube growth into the embryo sac is observed.

Arabinogalactan proteins are also expressed in ovule tissues and in the synergids, and have been suggested to act like signaling molecules or attractants for pollen tube guidance since they were detected by immunolocalization in ovules and synergid filiform apparatus of several species (Coimbra and Salema, 1997; Coimbra and Duarte, 2003; Coimbra et al., 2007). The most recent studies in Arabidopsis revealed the presence of specific AGPs in these tissues (Pereira et al., 2014), making it easier to further analyze their specific functions in the ovules. The presence of AGPs throughout different plant species is quite striking, even in the cork tree *Quercus suber*, recent studies, using immunolocalization as a detection method, revealed the specific presence of AGPs in the synergids and their filiform apparatus (Lopes et al., 2016).

## Pollen Tube Burst, Sperm Cell Release, and Polytubey Blockage: One Life Stand

Once in the ovule micropyle the pollen tube must enter the embryo sac through one of the synergids, arrest growth and burst inside it, releasing the two sperm cells. It was shown that the pollen tube does not enter directly into the synergids through the filiform apparatus but rather grows along this structure entering the embryo sac in a region with less cell wall invaginations (Leshem et al., 2013). In the recent years, the molecular interactions involved in pollen tube reception by the synergids as well as between the sperm cells and the female gametes have been thoroughly studied shedding some light into all these mechanisms (reviewed in Bleckmann et al., 2014). Although there is a long way to go until we comprehend all the mechanisms regulating such an important process for seed formation.

One of the first proteins to be related to pollen tube reception by the synergids was FERONIA/SIRÉNE (FER/SRN), a receptor like serine/threonine kinase that localizes to the synergids filiform apparatus (Huck et al., 2003; Rotman et al., 2003; Escobar-Restrepo et al., 2007). *fer/srn* pollen tubes fail to arrest growth, continue growing and do not release the sperm cells. This showed the active involvement of FER/SRN in mediating the signaling pathway responsible for pollen tube-synergid interaction. FER, as a *Catharanthus roseus* RLK1-like kinase (CrRLK1Ls) possesses a malectin-like extracellular domain, thus, its ligand could be a carbohydrate or glycoprotein from the cell wall (Lindner et al., 2012a). The search for more candidate molecules involved in this step identified LORELEI (LRE) as a glycosylphosphatidylinositol (GPI)-anchored protein, predominantly expressed in the synergid cells. *lre* pollen tubes present a similar phenotype to that of *fer*/*srn*, where the pollen tube enters the embryo sac but fails to arrest and release the sperm cells (Capron et al., 2008). LRE is localized in the plasma membrane, therefore being a perfect candidate to participate in pollen tube-synergid interactions (Tsukamoto et al., 2010). Recently, Liu et al. (2016) have proved that LRE and FERONIA jointly function in pollen tube reception at the interface of the synergid cell and pollen tube. Through an elegant study, it was shown that LRE has two independent functions. Pollen tubes ectopically expressing LRE rescue *lre* mutants, being essential for this step of reproduction. This complements a study showing LRE function as chaperoning FER localization in the filiform apparatus (Li et al., 2015). Liu et al. (2016) also revealed an important finding about LRE; that its GPI anchor is not entirely essential for LRE function. It will be extremely important to check if this also applies to other GPI-anchored proteins, such as AGPs or ENODLs. This may determine if these glycoproteins are acting attached to the plasma membrane or, if they are released by specific enzymes, to interact with other molecules located away from them. Another gene identified whose loss-of-function resemble the *fer* phenotype is *NORTIA* (*NTA*), which encodes the *MILDEW RESISTANCE LOCUS O 7* (*MLO7*) gene (Kessler et al., 2010). NTA localizes to cellular uncharacterized compartments, and becomes re-localized to the plasma membrane upon pollen tube arrival, in a FER-dependent manner, connecting FER to NTA in the same signaling network (Kessler et al., 2010). NTA contains a calmodulin-binding domain in its cytoplasmic C-terminus, possibly allowing synergid cell perception of Ca^2+^ oscillations during pollen tube reception (Iwano et al., 2012). Both *lre*/*lre* and *nta*/nta have a similar phenotype to *fer* but not fully penetrant like in the later, suggesting that they are important for pollen tube-synergid interaction but are probably acting redundantly with other unknown factor. *abstinence by mutual consent*, *amc*, also has a phenotype similar to *fer*, but this mutant is self-sterile, that is, the phenotype is observed only when both the pollen tube and the FG carry the *amc* allele (Boisson-Dernier et al., 2008). *AMC* encodes a peroxine involved in protein import in peroxisomes, which could be important for the production of small signaling molecule such as ROS or NO, being possibly involved in pollen tube-synergid signaling. *TURAN* (*TUN*), encoding an UDP-glycosyltransferase protein was also identified as a possible molecule involved in pollen tube reception, since its mutant as a *fer*-like pollen tube overgrowth phenotype (Lindner et al., 2012b). VERDANDI (VDD) is a transcription factor belonging to the REproductive Meristem (REM) family, and *vdd* shows defects in antipodal and synergid cell identity resulting in the absence of pollen tube burst so growth continues after targeting the synergid cells. Differently from the mutants described above, the overgrowth phenotype was not observed in *vdd*. VDD may act downstream of these cell surface signaling components (Matias-Hernandez et al., 2010). Recently, VDD and VALKYRIE (VAL), also a member of the REM family, were shown to act as a complex regulating the death of the receptive synergid cell, and the death (by bursting) of the pollen tube after growth arrest (Mendes et al., 2016).

Not so much is known about the male signaling components involved in pollen tube-synergid interactions. So far, ANXUR1 and 2 (ANX1 and ANX2) are the main components identified as pollen intervenients, they are closely related homologs of FER (Boisson-Dernier et al., 2009). The two RLKs are localized on the pollen tube tip plasma membrane and redundantly control the timing of pollen tube discharge. Their overexpression inhibits growth by over-activating exocytosis and over-accumulation of secreted cell wall material (Boisson-Dernier et al., 2013). Given this, the authors suggested that ANX inhibits pollen tube rupture and sperm discharge at the tip of growing pollen tubes constitutively, allowing pollen tube growth within maternal tissues, maintaining their cell wall integrity, until they reach the FG. Once at the FG, the female FER-dependent signaling cascade is activated to mediate pollen tube reception and fertilization, while male ANX-dependent signaling is deactivated, enabling the pollen tube to rupture and deliver its sperm cells (Boisson-Dernier et al., 2009; Miyazaki et al., 2009). Three pollen expressed transcription factors MYB97, MYB101, and MYB120, were reported to control the expression of genes whose encoding proteins play essential roles in pollen tube-synergid interactions (Liang et al., 2013). The single mutants did not reveal any phenotype, only the triple *myb97 myb101 myb120* pollen tubes revealed uncontrolled growth and failed to discharge sperm cells after entering the synergids. These transcription factors are critical for the pollen tube to exchange signals with the synergids (Liang et al., 2013).

Very recently, Hou et al. (2016) described a small group of ENODLs, EN 11 – 15, as essential for pollen tube reception in Arabidopsis. These glycoproteins, described in the section above, are considered to be chimeric AGPs. They are expressed in the ovules, and at least two of them, EN14 and EN15, are enriched in the synergid filiform apparatus. The quintuple mutant revealed severe pollen tube defect during its entrance into the embryo sac. In this mutant the pollen tube enters the embryo sac but continues to grow and does not burst, similar to what happens in *fer*. Actually, the authors have proved that at least EN15 interacts with FER, suggesting its involvement in the signaling pathway activation.

Iwano et al. (2012) pointed out a possible role for the secondary messenger Ca^2+^ in regulating sperm cell delivery and fertilization. Cytosolic [Ca^2+^] was shown to be essential for the control of PCD in both pollen tube and the receptive synergid and for sperm cell fusion with female gametes. Two different studies (Denninger et al., 2014; Ngo et al., 2014) showed that the pollen tube contact with the receptive synergid, mediated by FER and LRE, initiates oscillations in synergid cytosolic [Ca^2+^] signatures. These oscillations culminate in a change from [Ca^2+^] oscillations to a sustained global [Ca^2+^] flood in the receptive synergid, until the moment the pollen tube bursts leading to PCD of the receptive synergid, and pollen tube own PCD. NTA is proposed to modulate the intensity of the synergid [Ca^2+^] signatures in the synergids through its calmodulin binding site (Ngo et al., 2014). Ngo et al. (2014) proposed that the persistent synergid either retains its [Ca^2+^] signature, being responsible for repelling additional pollen tubes after successful double fertilization or reprograms its [Ca^2+^] signature to that of the receptive synergid upon the arrival of new pollen tubes for rescuing a failed fertilization event. Nevertheless, the signaling events responsible for PCD in the synergid cell are not understood yet. These Ca^2+^ oscillations may, as described in the above sections, be also regulated by AGPs acting as Ca^2+^ capacitors, given that they are abundant in pollen tubes and ovules.

In Arabidopsis, receptive synergid degeneration occurs after pollen tube arrival, but before pollen tube discharge (Sandaklie-Nikolova et al., 2007) suggesting a pollen tube derived signal to initiate synergid cell degeneration. About the persisting synergid PCD little information is available. According to Schneitz et al. (1995), the persistent synergid disappears during the second and third endosperm nuclear division.

Following pollen tube entrance into the embryo sac through the receptive synergid there is a rapid transport of the sperm cells by pollen tube discharge to the chalazal pole of the receptive synergid. Sperm cells adhere to the surface of the female gametes with no preferential order regarding their position in the male germ unit. After this, sperm cells remain immobile for a fairly long period, approximately 7.4 min, in the boundary between the egg and the central cell (Hamamura et al., 2011). Afterward, the membranes of both gametophytes fuse (plasmogamy) almost simultaneously, and the two sperm cell nuclei resumed their movement inside each female gamete toward their nuclei (Hamamura et al., 2011). During sperm cell fusion with the female gametes three central players have been identified so far: GAMETE EXPRESSED 2 (GEX2) (Mori et al., 2014); GENERATIVE CELL SPECIFIC 1/HAPLESS 2 (GCS1/HAP2) (Mori et al., 2005; von Besser et al., 2006) and the EGG CELL 1 (EC1) family (Sprunck et al., 2012). *GEX2* encodes a sperm expressed protein localized to its membrane, containing extracellular immunoglobulin-like domains, and is required for gamete fusion. In *gex2* sperm cells there is a reduced adhesion to female gametes, likely causing cell fusion failure (Mori et al., 2014). *GCS1/HAP2* is predicted to encode a protein with an N-terminal secretion signal, a single transmembrane domain and a C-terminal histidine-rich domain expressed only in the haploid sperm. *gcs1/hap2* sperm cells delivered to ovules fail to initiate fertilization, the released sperm cells remain at the fusion site with female gametes, and end up leading to the attraction of multiple pollen tubes (Mori et al., 2005; von Besser et al., 2006). EC1, small cysteine-rich protein, is predicted to activate sperm cell for gamete fusion (Sprunck et al., 2012). EC1 is stored in vesicles in the egg cell, and secreted to the apical region of the degenerative synergid cell conducting to re-localization of the fusogen GCS1/HAP2 to the cell surface of the sperm cell. Before fertilization GCS1/HAP2 is localized in the endomembrane system (Mori et al., 2005). After this, sperm cells are activated and ready to fuse with the female gametes, leading to double fertilization.

In order to prevent multiple fertilization events (polyspermy), that could lead to embryo lethality or malformation of the endosperm, and thus reproductive failure, plants evolved mechanisms to protect them from this risk.

Maruyama et al. (2013) showed that the successful fertilization of the egg and the central cell initiates a blockage mechanism mediated by the FIS-PRC2 (Fertilization Independent Seed – Polycomb Repressive Complex 2) complex, to avoid the attraction of multiple pollen tubes (polytubey). By the same time it was also shown the involvement of ethylene in triggering the persistent synergid cell death in order to avoid polytubey, involving the transcription factors EIN2 and EIN3 (ethylene insensitive 1 and 2). With the death of the persisting synergid, production of pollen tube attractants stop, leading to the polytubey blockage (Völz et al., 2013). It is now known that the persistent synergid cell is eliminated by its fusion with the endosperm and elimination of its nuclear contents: synergid-endosperm fusion (Maruyama et al., 2015). The same study revealed that this fusion is dependent on FIS-PRC2 complex and ethylene signaling activation by the central cell and the egg cell, respectively. Recently, Motomura et al. (2016) discovered that synergid-endosperm fusion takes place even in Arabidopsis mutants defective in MULTICOPY SUPRESSOR OF IRA1 (MSI1), a subunit of the PRC2 complex. In this mutant endosperm develops without the occurrence of fertilization. It reveals that the central cell fertilization is triggering the SE fusion, although synergid nuclear disorganization seems not to occur. This last step of the process needs to be clarified. It remains to be known how the persistent synergid nucleus is recognized as a target for elimination.

JAGGER (AGP4), the first specific AGP from Arabidopsis to reveal a function in the fertilization process, is important to prevent polytubey (Pereira et al., 2016b). *jagger* mutants fail to undergo persistent synergid cell death, leading to the attraction of more than one pollen tube per embryo sac. In this study it was demonstrated that JAGGER from the ovule integuments is the one responsible for this phenotype. How JAGGER is involved in this complex mechanism is still unknown. JAGGER might be one of the cell death markers for the persistent synergid nucleus, destining it for elimination (Pereira et al., 2016a). AGPs have already been described as molecular markers during development (Coimbra et al., 2007; Tucker and Koltunow, 2014).

In conclusion, this last step of pollen tube growth including pollen tube burst, sperm cell discharge and fusion with the female gametes along with the polytubey blockage system involves a complex and intricate system of communication and signaling mechanisms between the pollen tube and the female sporophytic and gametophytic tissues, ensuring double fertilization and, therefore, reproductive success.

## Conclusion and Outlook

Considering AGP roles, either as signaling or nutrient molecules and their carbohydrate epitopes differential presence in space and time along male and female reproductive tissues, it is unquestionable to affirm that AGPs are basal component interactors along the crosstalk channel established between the germinating pollen tube and the female tissues, for a successful double fertilization.

Arabinogalactan proteins can work as major players in the different steps of interaction between the pollen grain, pollen tube and the pistil tissues as they might intervene in pollen grain recognition, adhesion and germination and pollen tube growth initiation. AGPs are also fully developed to function as guiding, support, and nutrient providing molecules for pollen tube growth through the transmitting tract or mucilage of hollow pistils, along the style and the ovary and they are present in the ovules and funiculus, possibly working as attracting molecules for this final stage of pollen tube micropylar guidance. AGP presence at the ovule entrance tissues reveals their involvement also in the control of multiple pollen tube blockage – as evidenced by JAGGER (Pereira et al., 2016b).

Despite the need to use modern molecular tools to identify specific AGPs along the pollen tube growth pathway, immunolocalization studies of these glycoproteins are still essential to continue studying them. Not only because they are used to detect them in different plant species, but also because the specific glycosylations of each AGP is still not known. The available antibodies for immunolocalization studies are the only ones that detect different glycosidic AGPs epitopes. Different labeling patterns with different antibodies specific for the AGPs’ carbohydrate moiety may reflect differences in the types of glycosylations present at different cell types and developmental stages. Besides, the great diversity of carbohydrates decorating AGPs may implicate a broad range of affinity and specificity of these molecules for wide variety of plant developmental functions. On the other hand, we do not know if the differences in glycosylations are very noticeable. This is an exciting field of research, but it is also what makes it so difficult to study this family of proteins.

If AGPs might play so many roles in so many different tissues, these will be due to differences in their carbohydrate moieties? Different glycosylation machineries may be available in different tissues leading to different kinds of posttranslational modifications? This was, many years ago, suggested by Pennell et al. (1991), as the hypothesis that each cell or tissue type, somatic or reproductive, may have different glycosylation machineries.

Recent AGPs specific hydroxyproline-*O*-β-galactosyltransferases loss of function mutants, *galt2*, *galt3*, *galt4*, and *galt5* (Basu et al., 2013, 2015a,b) and *hptg1*, *hptg2*, and *hptg*3 (Ogawa-Ohnishi and Matsubayashi, 2015) revealed the importance of the carbohydrate moieties of AGPs for its function, as speculated before, making them strong candidates for mediation of cell-cell communication. Besides, a different study suggest AGPs to act as calcium chelators (Lamport and Várnai, 2012). It will be interesting to discover if some specific AGPs expressed along the reproductive tissues are acting in this way, taking into account that many of the reproductive events are dependent on calcium oscillations (Iwano et al., 2004, 2012; Michard et al., 2011). The problem of considering AGPs acting as calcium capacitors, is that we would be considering all AGPs to act in the same way. Why would the plant produce such a big family of glycoproteins, expressed differentially along the diverse plants tissues? It can be that AGPs are playing all these different roles simultaneously, considering that the family is so huge, it is quite possible that some are acting as calcium capacitors, and others in other more specific functions, such as JAGGER or AGP18.

The increasing supporting information on the importance of the carbohydrate moiety of AGPs for its functions (Cheung et al., 1995; Wu et al., 2000; Mizukami et al., 2016) together with the hypothesis that AGPs, even if closely related at the amino acid level, might play unique and different functions in different processes (de Graaf et al., 2003; Yang et al., 2007; Pereira et al., 2014), are being fused with the huge amount of knowledge on AGPs distribution throughout sexual reproductive tissues in many species. This will thus deeply clarify AGPs functions and mode of action, sustaining the fair importance these molecules are having in sexual plant reproduction.

## Author Contributions

AP, AL and SC made substantial contributions to the conception and design of this review, participated in drafting and revising it critically and give final approval of this version to be submitted and any revised version.

## Conflict of Interest Statement

The authors declare that the research was conducted in the absence of any commercial or financial relationships that could be construed as a potential conflict of interest.
